# Critical analysis of tobacco taxation policies in Pakistan after two decades of FCTC: Policy gaps and lessons for low- and middle-income countries

**DOI:** 10.18332/tid/173389

**Published:** 2023-11-24

**Authors:** Haleema Masud, Sharifah Sekalala, Paramjit Gill, Oyinlola Oyebode

**Affiliations:** 1 Wolfson Institute of Population Health, Queen Mary University of London, London, United Kingdom; 2 School of Law, University of Warwick, Coventry, United Kingdom; 3 Warwick Medical School, University of Warwick, Coventry, United Kingdom

**Keywords:** tobacco control, FCTC, tobacco tax, Article 6, tobacco pricing

## Abstract

**INTRODUCTION:**

Tobacco taxation remains a poorly used intervention to control tobacco use in many low- and middle-income countries (LMICs) including Pakistan even after two decades of FCTC adoption. This study identifies gaps and implementation challenges in the current Tobacco Taxation and Pricing Policies (TTPP) in Pakistan, and highlights key policy implications and lessons for LMICs to strengthen tobacco control measures.

**METHODS:**

We used qualitative document analysis to examine the policy documents to assess the TTPP against the WHO Framework Convention on Tobacco Control (FCTC) guidelines for the implementation of Article 6 of the FCTC. In addition, we used secondary data on tobacco tax and prices to assess the impact of TTPP on tobacco affordability in the country.

**RESULTS:**

Although Pakistan taxes raw tobacco, cigarettes and other tobacco products (cigarillos, cigars, cheroots), the existing TTPP falls below the WHO FCTC requirements of: uniform tax level, simple tax structure and 70% share of excise tax in the price of a product’s pack; among others. There are also multiple issues in tobacco tax administration such as lack of monitoring. This is leading to the availability of highly affordable tobacco products in the country.

**CONCLUSIONS:**

Pakistan does not have a clear strategy on using tobacco taxation and prices as a public health tool in the country. Existing TTPP face dual issues of flawed structure and poor administration translating into highly affordable tobacco products and low revenues in the country. There is a need to introduce multisectoral tobacco control policies in countries like Pakistan in the context of the tobacco sector political economy.

## INTRODUCTION

Year 2023 marks two decades since the adoption of the Framework Convention on Tobacco Control (FCTC), an international treaty to reduce the harmful impacts of tobacco consumption. It legally binds 182 countries, its parties to take action for tobacco control. Tobacco taxation and price increases are the most effective measures to control tobacco use. An increase in tobacco products’ prices leads to decrease in per capita consumption, promotes cessation, prevents relapse among quitters and more importantly deters people from starting tobacco use^1,2^. The World Health Organization’s (WHO’s) Framework Convention on Tobacco Control (FCTC) has recognized the importance of these measures and calls on the governments through Article 6 to implement tax and price measures to reduce tobacco use^3^. The Conference of Parties adopted detailed guidelines for implementation of Article 6 on its 6th Session^4^. Both high- and middle-income countries have successfully used these measures to reduce tobacco consumption in their countries^4-6^. The most recent evidence in this regard is from South Africa, where smoking prevalence reduced from 33% to 20% between 1993 and 2012 as a result of increase in excise duties of more than 500%^7^. Without such strict tax measures, the present number of smokers in South Africa could have been almost 4.5 million higher^8^.

Despite the proven effectiveness of tobacco tax and pricing policies (TTPP), the progress of implementation of the FCTC Article 6 is far from optimal as compared with the other tobacco control policies. A 2017 WHO report highlights this by giving average implementation rates of 88% for smoke free policies, 76% for advertising and sponsorship bans, and only 65% for TTPP^9^. Further, only 32 out of 188 countries have so far employed robust tax measures to control tobacco use^9^. The main challenge is for countries like Pakistan who grow tobacco and heavily depend on tobacco tax related revenues. The tobacco industry contributes almost USD 1 billion to Pakistan’s economy constituting up to 50% of the total federal excise duties (FED)^10^. Pakistan is signatory to the WHO FCTC and legally bound to control tobacco through evidence-based practices prescribed by the treaty. Article 6 of the WHO FCTC demands parties to implement TTPP ‘… *so as to contribute to the health objectives aimed at reducing tobacco consumption*’. TTPP is an under-researched area in Pakistan leading to a lack of evidence-based policy making^10^. This study identifies gaps in the current TTPP in Pakistan against the WHO FCTC requirements, and discusses the challenges in implementation of better TTPP.

## METHODS

We used qualitative document analysis (QDA) technique to analyze the policy documents, and secondary analysis of the price and affordability data to assess the impact of TTPP on affordability of tobacco products in Pakistan. The QDA was conducted in four steps: 1) development of the analytical framework, 2) finding and selecting policy documents, 3) the content analysis of policy documents; and 4) analysis of price data to assess the affordability of tobacco products.

We developed an analytical framework using the Article 6 of the FCTC (Price and tax measures to reduce the demand for tobacco) and the guidelines adopted for implementation of the Article 6. We carefully reviewed these guidelines to identify the benchmarks for the assessment of tobacco taxation policy and for tax administration mechanisms and control of illicit trade. Some additional assessment standards were also included based on the WHO’s Manual on Tax Administration (2021).

We reviewed tobacco taxation policies, laws and the Statutory Regulatory Orders (SROs) published in the official gazette of Pakistan to update legislation (up to December 2022). We searched these documents through official websites of the Federal Board of Revenue (FBR), Pakistan Tobacco Board (PTB), Tobacco Control Cell (TCC), and through discussion with policy experts. We used the official websites of the National Tobacco Control Cell (TCC), Federal Board of Revenue (FBR), Inland Revenue Enforcement Network (IREN), and Pakistan Tobacco Board (PTB) to extract the data on the overall tobacco regulation in the country. We also searched the official websites of the civil society organizations dealing with tobacco and/or tobacco taxation for relevant information including the Campaign for Tobacco-Free Kids (CTFK) Tobacco Control Laws’ website. The current government policies, Acts and laws pertaining to tobacco control and tobacco taxation were: the Finance Acts, 2006–2007 to 2022–2023 (for tobacco tax structure and rates); the Federal Excise Act, 2005 amended up to 22nd August 2022 and the Federal Excise Rules, 2005; the Sales Tax Act, 1990 amended up to 22nd August 2022 and Sales Tax Rules, 2006; Sales Tax Special Procedures Rules, 2007; the Customs Act, 1969 as amended up to 30th June 2022; the Customs Rules, 2000; the Cigarettes (Printing of Warning) Ordinance, 1979 and the Prohibition of Smoking in Enclosed Places and Protection of Non-smokers Health Ordinance, 2002. The text on the official websites of PTB and TCC was also analyzed to aid the interpretations of policy statements.

We reviewed all relevant policies with a specific focus on sections that were concerned with tobacco, tobacco products, or cigarettes. These sections were highlighted for thorough reading. In addition, general sections which were relevant and concerned with the licensing, registration, operation of the business, and tax administration in Pakistan were highlighted for further examination. The analytical framework served as a guide for identifying relevant sections. Each highlighted section was carefully read and analyzed to determine the extent of the match with the benchmarks set by the FCTC for TTPP. The strength of the match with each benchmark was then categorized into four categories, ‘full match’, ‘partial match’, ‘unclear’ (the benchmark provisions were there in the policy, but details were lacking to make the decision) or ‘not matching’ the benchmark. In addition, the data for each benchmark was summarized to synthesize the findings. Analysis was done through qualitative assessment of the text which focused on interpreting the meaning rather than identifying the presence or absence of keywords or word frequency counts. A summary of each policy document with relevant benchmarks in it was produced in addition to the original annotated documents. This audit trail was maintained to ensure the scientific rigor of the analysis process and to aid review and validation.

### Data analysis for tobacco affordability

To measure the impact of TTPP on tobacco prices and affordability, we analyzed available data on tobacco products’ pricing. Consumer prices for cigarettes, snus and tobacco for hookah were collected from the monthly statistics bulletin issued by the Pakistan Bureau of Statistics (PBS).

Affordability of tobacco products was measured using the Big Mac Index, the number of cigarettes packs one can buy for the price of one Big Mac^11^. We adapted this index for snus as number of packs of snus (20 g each) for the price of one Big Mac. We used the prices of cigarettes as reported in the WHO report on the global tobacco epidemic and Big Mac prices published by the Economist to calculate the index^9,12^.

## RESULTS

Results are structured in three parts; first an overview of the tobacco regulating bodies in Pakistan, secondly an assessment of the current TTPP and thirdly the impact of these TTPP on consumer prices and affordability of tobacco products.

### Tobacco regulating bodies in Pakistan

Pakistan regulates tobacco prices mainly through three Ministries: Finance, Health and Ministry of National Food Security & Research.

The Ministry of National Food Security & Research exercises its control through the Pakistan Tobacco Board (PTB). The board was established for promoting financial and economic stability in Pakistan using tobacco growth. The PTB Ordinance, 1968 states the PTB was established for *‘the promotion of cultivation, manufacture and export of tobacco and tobacco products’*^13^. The PTB mainly facilitates and oversees cultivation of tobacco and trade between farmers and purchasers.

The ministry of finance through the FBR and Customs department is responsible for taxation and pricing of tobacco products to generate revenues. It uses two types of tax on domestically produced tobacco products in Pakistan; the FED and the general sales tax governed under the auspices of the Federal Excise Tax Act, 2005 and the Sales Tax Act, 1990 respectively. For imported tobacco products, import duties are applicable under the jurisdiction of the Customs Act, 1969.

In contrast to the ministries of commerce and finance, the ministry of health operates with a clear aim to reduce tobacco consumption in the country. The national TCC established within the ministry of health, operates with an objective of decreasing tobacco prevalence in Pakistan using administrative, legislative and coordination measures for FCTC implementation^14^.

### Tobacco taxation and pricing policies

*Tax structure*

Pakistan taxes raw tobacco, processed unmanufactured tobacco, manufactured cigarettes and other tobacco products like cigars, cigarillos, cheroots. There are no excise or sale duties on raw tobacco in Pakistan. However, two types of taxes are levied on raw tobacco, one under the auspices of the PTB and the other by the provincial government of the province where tobacco is grown (Table 1). PTB and KPK charge cess (tax or levy) on the purchase of raw tobacco from all tobacco buyers. PTB uses this cess as its operational budget while the provincial cess is to be spent on the districts where tobacco is grown (KPK Utilization of tobacco cess rules, 2007). The aim is to utilize this cess for the development of tobacco growing areas and on activities to further develop tobacco production.

**Table 1:** Tax rates for tobacco products in Pakistan, 2022–2023

*Tobacco products*	*Tax*	*Type of tax*	*Tax rate (in PKR per kg)*		*Tax destination*
Raw tobacco	+	Federal tobacco cess^a^	Flue cured Virginia tobacco	6.00	PTB
			Dark air cured tobacco	3.60	
			White patta	3.00	
			Burley	5.00	
			Naswar, snuff, hookah, and other rustica tobacco and its products	3.00	
		Tobacco development cess^b^	Virginia	6.00	Tobacco growing district
			White patta/rustica tobacco	3.00	
			Snuff/naswar	2.50	
Unmanufactured tobacco^c^	+	Excise	390		National exchequer
Naswar, paan, snuff, khaini, and other SLT	-	-	-		-
Bidis	-	-	-		-
Cigars, cheroots, cigarillos and cigarettes of tobacco substitutes	+	Excise	Ad valorem excise of 65% of the RP, or PKR 10000 per kg, whichever is higher		National exchequer
		Sales	17% of RP (including excise duty)		
Cigarettes	+	Excise (Specific)	RP per pack <133.2	41.0 per pack	
			RP per pack >133.2	130.0 per pack	
		Sales	17% of RP (including excise duty)		
Imported cigarettes		Excise	Ad valorem excise of 65% of the RP, or PKR 130 per pack, whichever is higher		National exchequer
		Sales	17% of RP (including excise duty)		
Tobacco mixture in an electrically heated tobacco product	+	Excise (Specific)	5200		National exchequer
E-liquids for electric cigarette kits	+	Excise (Specific)	10000		National exchequer

PKR: 1000 Pakistani Rupees about US$3.5, current exchange rate. RP: retail price. SLT: smokeless tobacco products.

a Ministry of National Food Security and Research, SRO 868 (I)/2022 (https://ptb.gov.pk/sites/default/files/2022-08/SRO%20dated%2022-06-2022.pdf).

b The Khyber Pakhtunkhwa Finance Act, 1996 (https://www.kpexcise.gov.pk/app/tobacco-development-cess/).

c Tobacco useable for manufacture of cigarettes as manufactured by Green Leaf Threshing Units after processing and conversion of tobacco green leaf, Ministry of National Food Security and Research, SRO 868 (I)/2022 (https://ptb.gov.pk/sites/default/files/2022-08/SRO%20dated%2022-06-2022.pdf). Source: Federal Excise Act, 2005 as amended up to 22 August 2022 (https://www.fbr.gov.pk/Categ/Federal-Excise-Act/346 [last accessed 27/10/2022]).

There are no excise or sales taxes on bidis and smokeless tobacco (SLT) products. The Federal Excise Duty Act 2005, exempts unmanufactured tobacco that is used in tobacco products other than cigarettes and mixtures for pipes, cigars, and cheroots. Sales tax is not applied on products from cottage industries and small businesses with annual revenue <5 million PKR (current exchange rate: 1000 PKR about US$3.5).

**Figure 1:**
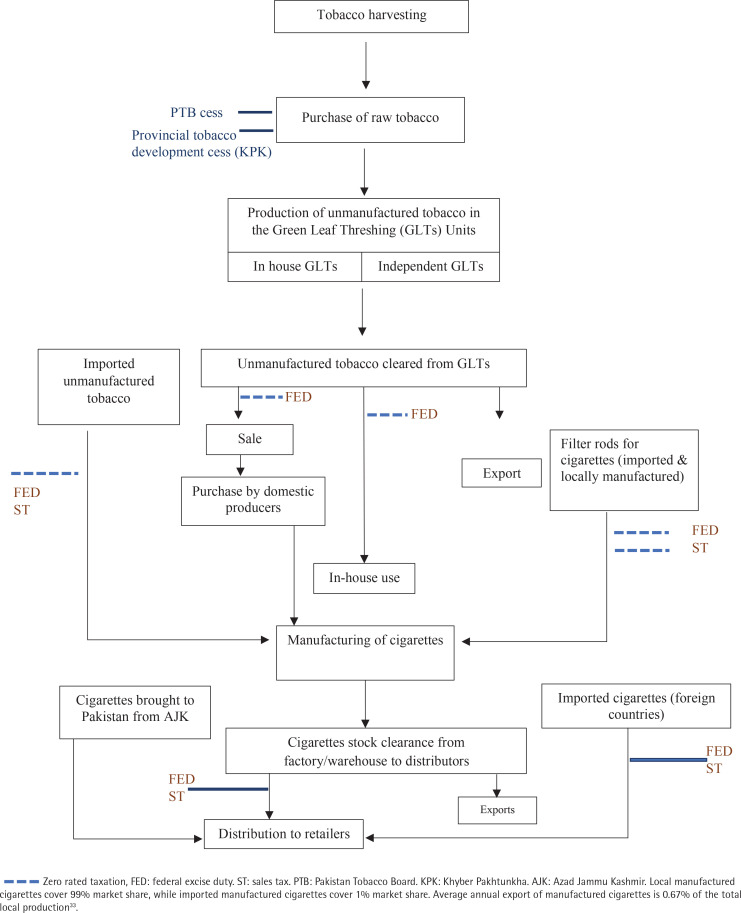
Taxation system for cigarettes in Pakistan

Pakistan applies both excise and sales tax to manufactured cigarettes at different stages of production and distribution (Figure 1). The current tax structure for cigarettes is complex as different rates are applied on two tiers of cigarettes. These tiers are based on retail prices (excluding sales tax), with lower tax imposed on lower price tiers. Currently, economy brand cigarettes (capturing ≥80% of the market share) are taxed at low rates of PKR 41.00 per pack. The average tax share in retail price for cigarettes is almost 44.3% as opposed to the minimum 70% benchmark set by the WHO.

Table 2 provides an overview of the tobacco tax structure in Pakistan against FCTC guidelines and the best practices recommended by the WHO^15^. It highlights that Pakistan does not meet these benchmarks in most respects (uniform tax structure, minimum tax level, taxation of all tobacco products, decreasing affordability).

**Table 2:** Assessment of tobacco product taxation structure against the FCTC guidelines for the implementation of Article 6

*Benchmarks*	*Analysis base*	*Assessment*	*Comments*
**Strategic level policy**			
Include tobacco taxation as part of a comprehensive tobacco control program	Policy documents related to tobacco control, official websites of tobacco control cell, tobacco board, the Ministry of Finance and Ministry of Health	C	Currently the FBR documents do not mention tobacco taxation as a tobacco control tool. However, the TCC has been continuously advocating and liaising with the FBR to increase tobacco taxation to control tobacco use in the country.
TTPP should be designed in a way to reduce affordability of tobacco products over time in order to reduce consumption and prevalence		C	The FBR documents on tobacco taxation do not make a commitment on reducing the affordability of tobacco products. In June 2017, cigarette prices were markedly decreased from PKR 70 to PKR 48 for the most commonly sold and other economy brands, indicating no such commitment.
Need to have long-term policies on tobacco tax structure to achieve public health, fiscal and other objectives		C	There are no short- or long-term policies on tobacco taxation to achieve public health goals.
When deciding on TTPP, take into account both the price elasticity and income elasticity of demand, as well as inflation and changes in household income		B	The FBR has never considered price elasticity or income elasticity as well as income growth in deciding on tobacco taxation. However, historical account shows that price increase was keeping pace with inflation.
The TTPP should be protected from commercial and other vested interests of the tobacco industry as well as from any other actual and potential conflicts of interests		C	There are no policies to protect against potential conflict of interests and media reports show an influence of tobacco industry on the FBR’s decisions on tobacco taxation.
**Tax level and structure**			
Tobacco excise tax levels should be at least 70% of the retail price for tobacco products	Finance Acts (2013–2018)	C	Current tax share of the leading economy brands is 41% (which capture more than 80% of market share). However, after the 2018 budget, tax share for the premium brands has met the benchmark for the first time (Figures 1 and 2).
Parties should implement the simplest and most efficient system that meets their public health and fiscal needs		C	Pakistan applies a tiered taxation regimen for cigarettes which is complex, less efficient and does not meet the public health objectives.
Increase tobacco taxes by enough to reduce the affordability of tobacco products	Affordability analysis of price data	B	The affordability of cigarettes had been decreasing from 2012–2016 but they became more affordable in 2017. The 2018 budget has minimally increased the prices thus maintaining the affordability.
Automatically adjust specific tobacco taxes for inflation	SROs by FBR, Finance Acts (2013–2018), the Federal Excise Act, 2005	B	There is no particular policy to adjust taxes for inflation, however, historical account shows that inflation adjusted prices had been increasing till 2016, after that the price was drastically reduced (for economy brand, PKR 70 to PKR 48).
Tax rates should be monitored, increased, or adjusted annually (considering inflation and income growth) in order to reduce consumption of tobacco products	Finance Acts (2013–2018)	C	Although tax rates are adjusted annually in Pakistan, the motivation for this adjustment is revenues not the aim of reducing affordability or consumption.
Parties should consider implementing specific or mixed excise systems with a minimum specific tax floor	Finance Acts (2013–2018)	A	Pakistan is using specific excises for all domestically produced cigarettes (meeting over 95% of market share), but ad valorem tax on imported products.
**Similar tax burden on all tobacco products**			
Apply comparable excise tax on all brands of given tobacco products	Finance Acts (2013–2018), the Federal Excise Act, 2005	C	Pakistan has a tiered tax structure of cigarettes where it applies differential excises on cigarettes, lower tax on economy brands compared to premium brands, thus keeping them affordable.
Decrease price gaps between products (consider the use of Minimum specific excise floor)	Finance Acts (2013–2018), the Federal Excise Act, 2005	C	Pakistan has huge gap in prices of cigarettes, ranging from PKR 40 to 140 for a pack of 20 cigarettes. The minimum price law allows the price gap to be 45%.
Adopt comparable taxes and tax increases on all tobacco products	Finance Acts (2013–2018), FCTC compliance reports by Pakistan	C	Pakistan does not tax locally manufactured smokeless tobacco products, bidis, and smoking mixtures for huqqa (hookah) at all. Tax rate is comparable for premium brand cigarettes, cigars and cigarillos.
**Tax and duty-free sale**			
Prohibit or restrict (eliminate*) tax and duty-free sales of tobacco products	Federal Excise Duty Act, 2005	C	All tobacco products are exempted from taxation if supplied for consumption to Pakistan Navy or to the President of Pakistan, the President of Azad Kashmir, the Provincial Governors and their families and guests, and to the duty-free shops. Duty-free products are often available in local markets as well.
**Use of revenues for tobacco control**			
Consider using revenues to fund tobacco control program	The Federal Excise Act, 2005, Tobacco board website	C	A health levy was proposed in the Finance bill 2018 but was not approved. The provinces charge some cess on raw tobacco which is utilized on the development of tobacco growing districts. PTB charges cess to facilitate further tobacco growth and research.

A: meets the benchmark. B: partly meets the benchmark. C: does not meet the benchmark. Assessment is made with legislation updated till December 2022. Cess: tax or levy.

*Tax administration mechanisms*

The FBR collects excise and sales tax on tobacco products at a single point from the manufacturers. Tax is collected on a monthly basis for the stocks supplied to market. Manufacturers are required to issue a combined tax invoice (sales tax and FED) when the stock leaves their premises and obliged to pay the following month. There are many weaknesses in the tobacco tax administration mechanism in Pakistan against the FCTC guidelines and WHO’s benchmarks; a major weakness being reliance on tobacco industry data for measuring tax liability and lack of surveillance and monitoring mechanisms (Table 3).

**Table 3:** Pakistan’s tobacco tax administration mechanisms against the FCTC guidelines for the implementation of Article 6

*FCTC Guidelines for tobacco tax administration (Benchmarks)*	*Assessment*	*Comments*
**Authorization or licensing or control systems**		
**Transparent licensing/registration systems or equivalent control systems should be in place to relevant entities for the control of the supply chain**		
Tobacco growers	C	
Manufacturers and importers of tobacco products	A	All manufacturing companies and importers need to be registered for the purpose of taxation.
Manufacturing equipment	C	Tobacco companies are obliged to declare details of equipment and their capacity but there is no licensing requirement.
Distributers of tobacco products or manufacturing equipment	C	There is no licensing but the distributers of cigarettes are required by law to carry the excise and sales tax invoice issued by the manufacturers when transporting cigarettes (IREN, 2017).
Wholesalers and Retailers	B	A process of licensing of the retail outlets is started in the capital and few more cities under the Punjab Tobacco Vend Act, 1958.
**Warehouse system/movement of excisable goods and tax payments**		
Adopt and implement systems of warehouses to facilitate excise controls on tobacco products	A	All manufacturers are required by law to have a separate storage place in their premises for storing excisable goods (Federal Excise Rules, 2005; Rule 16).
Excise taxes should be imposed at the point of manufacture, imports or release for consumption from the storage or production warehouses	A	Both excise and sales taxes are imposed at the stage of release of stock from production warehouses for locally produced products while at importation stage for imported varieties (Federal Excise Rules, 2005; Rule 11).
Tax payments should be required to be made at fixed intervals or on a fixed date each month	A	Tax is paid on monthly basis and all manufacturers should pay it by the 15th of the following month for the stock which is moved out from the warehouses (Federal Excise Act, 2005).
Tax payments should include reporting of production and/or sales volumes, and price by brands, taxes due and paid, and may include volumes of raw material inputs	A	The sales and excise tax returns form demands the tobacco industry to report both the volume and value of the goods supplied in a specific period, as well as tax due and paid.
Tax authorities should allow for the public disclosure of the information contained within these reports through the available media, including those online	C	Information is not publicly available.
**Anti-forestalling measures**		
Implement measures to restrict the release of excessive volumes of tobacco products immediately prior to a tax increase	C	There are no specific anti-forestalling measures but cigarette manufacturers are not allowed to reduce their retail prices from the level which was adopted on the day of the last budget announcement. Furthermore, they are not allowed to introduce lower price variants in the existing brand family (Finance Act, 2017).
Measures to levy the new tax on products already produced or kept in stock, including those in retail (known as a floor-stock or inventory tax)	C	
**Fiscal marking to monitor production and imports of tobacco products**		
Use fiscal markings (such as tax stamps, banderols, or digital tax stamps) to distinguish legal tax paid products from illegal tax evaded products.	C	None of such technology is currently adopted. However, a debate is ongoing for implementing tobacco track and trace system since 2005. Non-tax-paid cigarettes are very common in the country holding a market share of 9% (Ross et al.22) to 35% according to a tobacco industry-funded research (Oxford Economics, 2017).
Consider the implementation of track and trace systems for tobacco products in line with Article 15 of the FCTC	C	
**Enforcement**		
Tax authorities should have the authority and capacity to conduct investigations, search, seizure, retention and disposal activities	A	Federal Excise Duty Act, 2005 gives authority to its designated officials to conduct search, investigations, seizure, retention and disposal activities.
Different enforcement agencies should share information	C	There is no formal transparent channel to share information between different authorities.
Appropriate range of penalties for non-compliance with tax law should be introduced such as suspension or cancellation of license or the application of more stringent conditions on the license, fines and/or jail, forfeiture of products, forfeiture of equipment used in the manufacture or distribution of products including machinery and vehicles, cease and desist orders	A	Cigarettes manufactured in non-compliance with taxation laws are required to be confiscated by law along with any conveyance carrying those cigarettes (Section 26; Federal Excise Act, 2005). Counterfeit cigarettes are required to be destroyed by law (Section 27; Federal Excise Act, 2005). Section 19 of the Act also recommends a penalty with imprisonment for non-compliance with the Act and also seizure of any machinery and material involved. However, cancellation of registration is not a prescribed penalty.
Put in practice the penalties for late payment including interests	A	There are penalties for late payment in the law but these are quite soft. In case of non-filing, a fine of PKR 5000 ($41; 2005 exchange rate) is required along with the due payment. In case of short payment PKR 10000 ($82) or 5% of the duty involved (whichever is higher) in addition to the due payment (Section 19; Federal Excise Act, 2005).
Penalties for non-payment may include back taxes, punitive taxes		
Additional benchmarks as per WHO’s technical manual on tobacco tax administration*		
Tobacco excise department: A tobacco excise department should be established to deal with all matters related to tobacco taxation	C	There is no special tobacco excise department.
Tax authorities should be able to assess production levels and accurately estimate manufacturers’ tax liabilities, independent of claims filed by tobacco manufacturers	C	FBR relies on industry data which they report while paying taxes. There is no mechanism to assess actual production levels and tax liabilities.
Tax authorities should audit taxpayer account books periodically	B	Law allows FBR to do so, however, it is not a common practice. A recent audit is under process as directed by the Public Accounts Committee.
Knowledge/data management: Tobacco excise department should also maintain and update a comprehensive database for use in assessing tobacco product markets, conducting analyses of demand for tobacco products, evaluate the impact of tobacco tax increases and evaluating current tobacco excise taxes and the impact of increases in these taxes	C	There are no comprehensive data and FBR relies on the industry. A recent example is reliance on industry-based illegal market share data. Tobacco excises, being the top contributor of FED are officially reported in the revenue division year books and FBR seems to adjust tax rates based on last year’s revenues.

A: meets the benchmark. B: partly meets the benchmark. C: does not meet the benchmark.

* Benchmarks prescribed by the WHO’s technical manual on tobacco tax administration alone and not a part of the FCTC guidelines. Assessment is made with legislation updated till December 2022.

*Price control measures and regulations*

Pakistan regulates cigarette prices using the minimum retail price laws and bans on price promotions. The law prohibits the sale of tobacco products below market value, free samples, free goods and cash rebates [SRO 53(KE)/2009]. However, implementation of such bans is poor. According to the Global Youth Tobacco Survey, almost 10% students had been offered a free tobacco product^16^. Price discounts, other promotional offers and free gifts are also common^17,18^.

The minimum retail price law currently requires cigarettes to be priced at a rate which is not below 45% of the price limit specified for highest tier brands. However, it is not uncommon to find cigarettes being sold even at a quarter of this minimum limit^17^. FBR admits that there are no mechanisms to ensure application of this minimum price at the retail level^17^.

### Impact of TTPP on prices and affordability of tobacco products

*Cigarettes*

**Figure 2:**
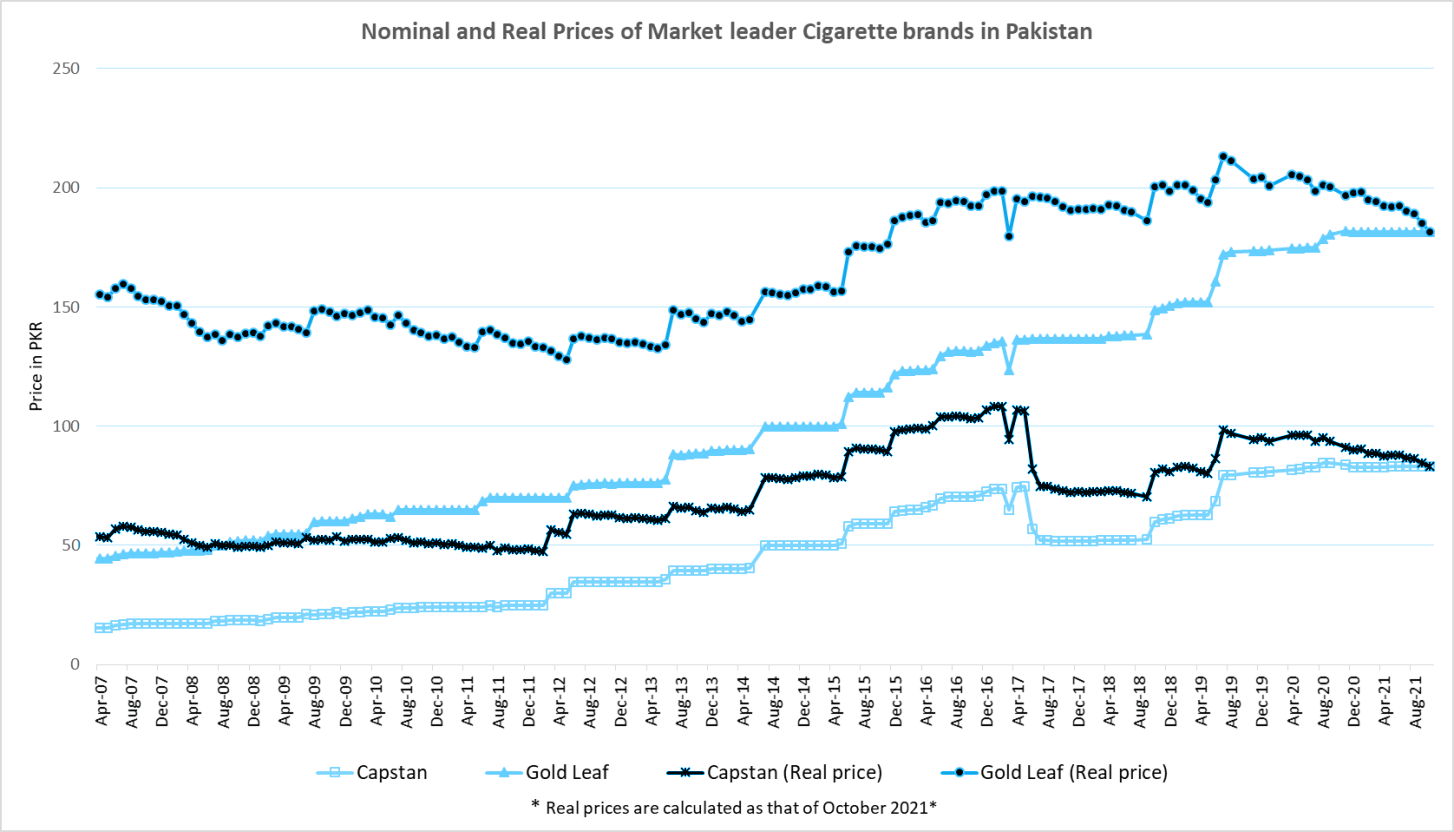
Trends in nominal and inflation adjusted consumer prices for market leader economy and premium cigarette brands (source Pakistan Bureau of Statistics-monthly statistical bulletin)

The average nominal consumer price for the premium brand (Gold Leaf) cigarettes increased consistently from PKR 44.16 (in Jan 2007) to PKR 181.63 (in October 2021), a rise of over 300% while the average real price has increased by only 24%. The inflation adjusted price has recently decreased from PKR 203 (in June 2020) to PKR 181.6 (in October 2021). The real price of economy brands increased by 52% from 2007 to 2021 in contrast to the nominal price increase of over 400%. The trend of price change is depicted in Figure 2.

Currently cigarettes are highly affordable in Pakistan. One can purchase more than 8 packs of the market leader brand of cigarettes for the price of one Big Mac in Pakistan (https://www.economist.com/big-mac-index). Cigarettes have become more affordable over last year with a Big Mac Index of 6.99 in 2021, and 8.43 in 2022.

*Snus (naswar)*

Average nominal price of 250 g of snus was PKR 64.24 in October 2021 which rose by almost 13% from November 2019, while the real price has decreased by 3% over the same period. On average, one can purchase 2257 g of naswar for the price of one Big Mac (1000 PKR about US$3.5; current exchange rate)^1^ in Pakistan. This translates into 112 packs of snus (20 g each).

*Tobacco for hookah*

The price for 1 kg of tobacco for hookah was PKR 239.91 in October 2021. The inflation-adjusted price has remained almost at the same level since November 2019 (PKR 239.17) while the nominal price has shown an increase of 17.7% from PKR 203.81 to PKR 239.17 from November 2019 to October 2021.

## DISCUSSION

This analysis shows that existing TTPP in Pakistan do not meet WHO FCTC requirements for implementation of Article 6. The TTPP face dual issues of flawed structure and poor administration translating into highly affordable tobacco products and low revenues in the country.

Pakistan’s TTPP keep tobacco products affordable for all income groups in the country. Pakistan has 23.9 million tobacco users, of which 10 million use SLT and 3.7 million use hookahs (waterpipes). However, TTPP focus only on cigarettes. Lack of taxation on SLT products has led to extreme affordability. One can purchase 112 packs of 20 g snus for the price of one Big Mac. This is cheaper than the cost of a loaf of bread (chapatti) or a cup of tea in the country. The main challenge in this regard is the informal and unregulated SLT industry. A possible way forward would be registration and licensing of SLT manufacturers, bringing them into the tax net. Cigarettes, too, are extremely affordable; one can purchase almost 8 packs for the price of one Big Mac while this figure was under 2.5 packs in 2012^19^. Pakistan has one of the cheapest cigarettes in the region, the 2020 prices of the most sold brand in international dollars at purchasing price parity (PPP) in Pakistan was $2.06 which was lower than the prices in Iran $6.01, India $8.66, and Bangladesh $2.91^6^. Considering that cigarettes have become significantly less affordable in many countries, this trend in Pakistan is alarming^19^. Worse, illicit cigarettes are often sold at one-quarter of the price of legal cigarettes^18^. This can be attributed to flawed tax structure and poor monitoring.

The tiered structure of cigarette taxation provides an opportunity for smokers to switch to cheaper brands if they have a decrease in affordability. The tax floor for economy brand cigarettes is only PKR 41 per pack. Although the introduction of uniform excise tax and thus abolition of the tiered system is the ideal solution, increasing the tax floor could be a feasible short-term solution.

Reforms in tax rates or structures alone cannot be effective in reducing the affordability unless coupled with strong administrative changes and effective monitoring. An example of a failure of the increase in tax rates alone can be seen in Pakistan between 2013–2016. Where, the tax increases led to high priced legal cigarettes but also to an increase in the illicit market and decreased government revenues^20,21^. The tobacco industry successfully convinced the FBR that higher taxes had led to increased illicit tobacco market leading to a reintroduction of the third tax tier and a marked decrease in cigarette prices in 2017^22^. The track and trace system was recently introduced in the country. However, it is not fully implemented yet. There are still concerns that a lack of field monitoring mechanism can cause failure of such system. Use of advanced technology to monitor production and movement of tobacco products is one solution to this, as was successful in Kenya where implementation of modern track and trace systems resulted in markedly reduced illicit market share and increased government revenue^23^. The introduction of such a system in Pakistan has been debated since 2005^24^. While waiting for implementation of this system, Pakistan could use various existing data from the manufacturing flow of cigarettes (Figure 1). A key step is the manufacture of processed tobacco from raw leaves in GLT units. Currently 10 GLTs operate in Pakistan and all cigarettes manufactured in Pakistan, and Azad Kashmir uses these units^25^. The Government of Pakistan, FBR^26^ through SRO 1149(I)/2018 has recently introduced a law to monitor the GLTs through the Inland Revenue department. Strict monitoring of these units could be helpful in cross-checking the quantities reported by the manufacturers. The quantity of filter rods (for cigarettes) manufactured can also be used to cross-check cigarette production.

### Policy implications and lessons for LMICs

*Need for national level policy on using taxation as tobacco control measure*

Underlying the identified issues is a lack of national level strategic framework for using TTPP for tobacco control. The WHO FCTC guidelines on implementation of Article 6 demand governments to have explicit long-term strategic plans with numerical targets. Countries like UK, NZ and Australia have set targets for annual increases in taxation of tobacco products by 2%, 10% and 12.5% above inflation, respectively^27-29^. However, no such objective policy is seen in Pakistan, not even a commitment to annual increases in tax rates. This can be attributed to the inherent conflict and competing interests between different government departments. While the Ministry of Health promotes higher tobacco taxation to reduce consumption, the Ministry of Commerce is operating to promote tobacco growth and manufacturing to bring economic and financial stability. Meanwhile, the Ministry of Finance is ‘addicted’ to huge revenues from cigarettes. One cannot ignore the fact that cigarettes contribute almost 50% of the total FED in Pakistan. To improve population health, the government of Pakistan needs to be clear about its stance on using TTPP for tobacco control as one unit, rather than different ministries operating in conflict with each other. Without deliberate action by the government of Pakistan to decrease affordability of tobacco products, their use is likely to remain highly prevalent affecting the lives of millions of people in the country.

*Need for coordination among different government departments*

A further issue is the lack of coordination among relevant enforcement bodies, such as the Inland Revenue department of the FBR, the PTB, TCC, District Food Authorities, police departments, provincial and district health departments. The district authorities responsible for implementation of other tobacco-related laws or provincial food authority inspectors should be coordinated with the FBR Inland Revenue department to implement minimum price laws. Coordination between the Tobacco Vendors Act implementation and the Inland Revenue department could keep track of the market to capture the illicit market at the retail level and help with implementation of the minimum sale price law. The smokeless tobacco products can also be brought in tax net using such coordination. Such district level coordination among different staff like health, education, police, food and municipal officers in Karnataka has been proven successful in implementing tobacco control laws^30^.

*Tobacco industry interference*

One cannot ignore the possibility of tobacco industry interference contributing to poor TTPP in Pakistan. The tiered tax structure with low excise duties has resulted from continuous lobbying from the tobacco industry^24,25^. The delay in introducing tobacco track and trace systems is also attributed to them^18^. Tobacco industry interference is prevalent in almost every country. However, there are examples on how this can be controlled, e.g. in Thailand the tobacco control advocates used multiple strategies to prevent and counter tobacco industry interference in policy making^31^.

*Strengthen enforcement and use existing laws and resources*

Lack of effective administration and law enforcement undermines the effectiveness of TTPP. Although a minimum cigarette price law exists in the country, the FBR lacks resources and mechanisms to monitor its implementation at retail level^10,17^. This results in availability of low-priced tax evaded cigarettes. A similar case is observed in Malaysia where poor control of cheap illicit cigarettes has undermined the impact of minimum price laws^32^. A possible solution in this regard is using the district administration and Tobacco Vendors Act as highlighted above. Laws on price promotions and sale of loose cigarettes need to be strongly enforced to achieve the results from existing policies.

*Understanding the political economy of tobacco taxation in the country*

Tobacco taxation is deeply rooted in the political economy of countries given its economic contributions. Although the health and economic benefits of tobacco taxation are well known and policy guidelines are available to achieve the benefits, the political economies of countries pose a challenge in using the policy benchmarks. It is important to understand who gains or who loses from specific policies and how different actors influence policies to protect their interests.

## CONCLUSIONS

Pakistan does not have a clear strategy on using tobacco taxation and prices as a public health tool in the country. Existing TTPP do not meet the WHO FCTC requirements. The TTPP face dual issues of flawed structure and poor administration translating into highly affordable tobacco products and low revenues in the country. Without deliberate policy action to decrease affordability of tobacco products, their use is likely to remain highly prevalent affecting the lives of millions of people in the country. We have identified key steps that can help countries like Pakistan to move forward in using tobacco taxation as a tobacco control tool.

## Data Availability

The data supporting this research are available from the authors on reasonable request.
